# Determination of Clomipramine using eco-friendly solid-contact ionophore-doped potentiometric sensor

**DOI:** 10.1186/s13065-023-00938-x

**Published:** 2023-03-25

**Authors:** Adel M. Michael, Amr M. Mahmoud, Nesma M. Fahmy

**Affiliations:** 1 Pharmaceutical Chemistry Department, Faculty of Pharmacy, Ahram Canadian University, October city, Egypt; 2 Analytical Chemistry Department, Faculty of Pharmacy, Cairo University, Cairo, Egypt

**Keywords:** Clomipramine, Point-of-care, Green chemistry, Supramolecular chemistry, Calix[4]arene, Graphene nanoparticles, Solid-contact ion-selective electrodes

## Abstract

**Introduction:**

Clomipramine is a tricyclic antidepressant acting as a serotonin reuptake inhibitor. Its maximum plasma concentration (C_max_) is 13–310 ng/mL, the therapeutic range is 220–500 ng/mL and its toxic effect appears in doses above 900 ng/mL.

**Objectives:**

The fabrication of eco-friendly solid-contact ion-selective electrodes to evaluate the concentration of Clomipramine in different matrices based on disposable screen-printed carbon electrode.

**Methods:**

Disposable screen-printed carbon electrode was utilized as a substrate to fabricate the proposed sensors. The sensors were optimized to determine Clomipramine using calix[4]arene as an ionophore into PVC polymeric membrane to enhance selectivity towards the target analyte. The solid-contact sensor potential stability was improved by the incorporation of graphene nanoparticles transducer layer.

**Results:**

The sensors were assessed as per the IUPAC recommendations. The linearity range was 1 × 10^− 2^ to 1 × 10^− 5.3^ M. The sensors were successfully applied to determine CLM in the pharmaceutical formulation. Furthermore, the ion selective electrodes were applied for Clompiramine assay in spiked plasma for the purpose of Point-of-Care testing to be a diagnostic tool for therapeutic monitoring of the cited central nervous system agent. The findings were statistically compared to the reported method showing no statistically significant difference.

**Conclusion:**

This work was concerned with developing a green analytical method for the determination of Clomipramine. The proposed SC-ISE was mixed with graphene nanocomposite transducer interlayer. The graphene layer succeeded in preventing the formation of an aqueous layer so resulted in a stable, reproducible standard potential besides the rapid response time.

**Supplementary Information:**

The online version contains supplementary material available at 10.1186/s13065-023-00938-x.

## Introduction

Nowadays health and environmental protection is of increasing importance. Green Chemistry was launched to provide safer methods for health and the environment. This was obtained by using safe and low risk potential solvents [1]. And also controlling the produced waste products or carrying out waste treatment [2]. Several techniques were developed to evaluate the greenness of the method as analytical Eco-Scale [3], Green Analytical Procedure Index which is abbreviated GAPI [4], Analytical greenness calculator; AGREE [5, 6]. Electrochemical methods are considered inherently green techniques through the use of water and buffers as the main solvents. Moreover, potentiometric measurements are time and cost-effective, besides being simple with very few sample pre-treatment steps [7–10].

**Fig. 1:**
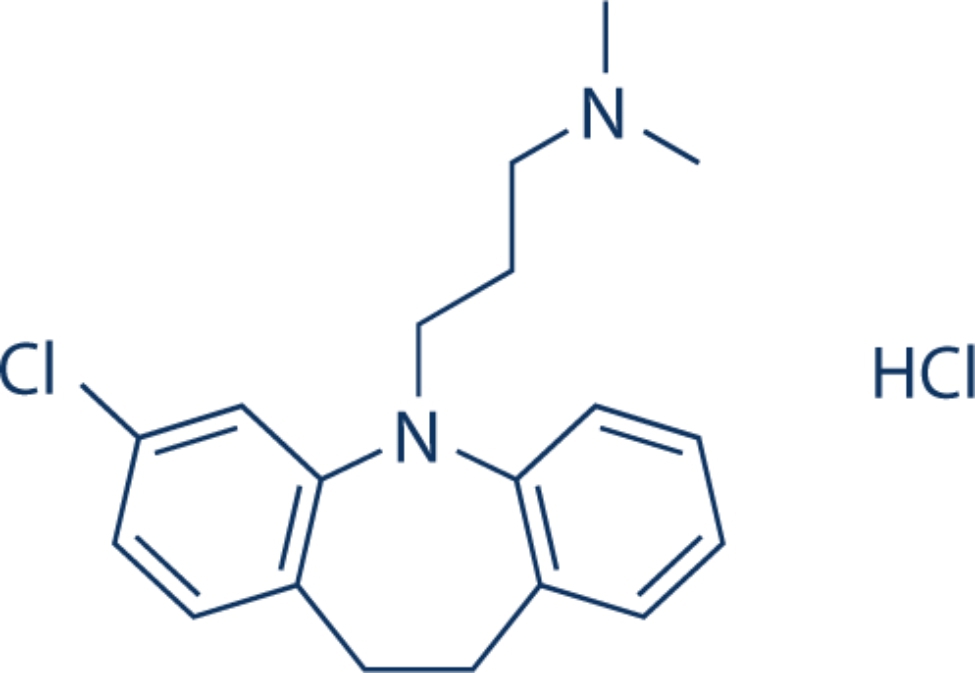
Chemical structure of Clomipramine HCl

Clomipramine HCl is a tricyclic antidepressant acting as a serotonin reuptake inhibitor [11]. Its chemical name is 3-(3-Chloro-10,11-dihydro-5 H-dibenzo[b,f]azepin-5-yl)-N,N-dimethylpropan-1-amine hydrochloride and the chemical structure is shown in Fig. 1. It is used in adults to treat symptoms of obsessive-compulsive disorder (OCD), depression, anxiety, and some eating disorders. It can be used as an adjunct for neuropathic pain as well as the treatment of premature ejaculation [12]. It has been approved for treating children above ten years old with OCD [13], attention deficit hyperactivity disorder (ADHD) and paediatric nocturnal enuresis [14]. Its maximum plasma concentration (C_max_) is 13–310 ng/mL [15]. The therapeutic range is 220–500 ng/mL and its toxic effect appears in doses above 900 ng/mL. Detecting low concentrations of CLM in biological fluids is crucial as to avoid toxicity or severe side effects such as blurred vision, urinary retention, and cardiac problems [14].

The literature revealed that CLM has been determined by spectrofluorimetry [16], and spectrophotometry [17]. Moreover, it was determined in biological fluids by immunoassays in urine [14], and by chromatography in serum [14, 18]. Electrochemical methods of analysis include potentiometric titration stated in the British pharmacopoeia (BP) [11], direct potentiometric assay by carbon paste electrodes [19, 20] and voltammetry [21].

The developed sensors have many advantages over the methods in the literature. Firstly, to improve the selectivity and sensitivity of ISE; the PVC membrane was doped with an ionophore [22]. The ionophore binds the target ion or form a complex with it to assure the transfer of the ion under investigation form the aqueous sample solution to the lipophilic membrane. This is obtained by decreasing the Gibbs free energy of transfer and thus results in increasing the sensitivity [23]. ISE widely uses ionophores as (2-hydroxypropyl)-β-cyclodextrin (2-HP-β-CD) and calixarenes (CX) as they have a 3D basket, cup or bucket structure [24]. The lipophilic core in their structure results in inclusion complex with selective attachment to the target ion [25]. They are utilized in ion-selective electrodes, selective membranes, as well as stationary phases [26] and have been widely used in electrochemical sensing [27, 28]. Secondly, Graphene (GR) is a 2D layer of graphite showing high hydrophobicity, chemical stability and superior electronic and electrochemical properties [29]. It is widely used in fabricating biological and electrochemical sensors [30, 31]. The inclusion of graphene inside polymers as a nanocomposite increases their electrical conductance [32, 33]. It can be prepared by solution casting where graphene is dispersed in organic solvent and then mixed with the polymer [34, 35]. Last but not least, the proposed method was proven to be eco-friendly. The method’s greenness was evaluated by Analytical greenness calculator; AGREE and GAPI approach.

The aim the current work was the fabrication of a green solid-contact ion-selective electrode (SC-ISE) with high accuracy and sensitivity to quantify CLM in the dosage form and plasma. To achieve that calix[4]arene was impregnated in the PVC polymeric membrane as the ionophore, and graphene was used as interlayer between the ion sensing membrane and the screen-printed carbon electrode as capacitive ion-to-electron transducer. The fabricated sensor was characterized and used to determine CLM in pharmaceutical formulation and spiked human plasma.

## Materials and methods

### Chemicals and materials

Clomipramine pure powder was supplied by Novartis Pharmaceutical Company, Cairo, Egypt, with purity 99.55 ± 0.89%. Anafranil^®^ tablets manufactured by Novartis pharmaceutical company (Egypt) were obtained from the Egyptian market claimed to include 25 mg Clomipramine HCl per tablet. Potassium tetrakis(4-chlorophenyl)borate (KTpClPB), calix[4]arene, and *o*-nitrophenyl octyl ether (*o*-NPOE) were bought from Sigma Aldrich (Missouri, United States). Polyvinyl chloride (PVC) was bought from Fluka (Steinheim, Germany). Graphene nano-platelets (6–8 nm thick × 5 microns wide) were obtained from Strem Chemicals INC. (Newburyport, USA). Britton – Robinson (BR) buffer was prepared by mixing phosphoric acid (0.04 M), acetic acid (0.04 M) and boric acid (0.04 M). Buffer solutions of different pH values were adjusted by the addition of 0.2 M NaOH. Screen-printed carbon electrodes (C-SPE) showing a diameter of 3 mm was obtained from CH Instruments, Inc., (Texas, USA).

### Instrument

Potential measurements were conducted using Jenway digital pH / mV meter - model 3505 (mad in UK) in combination with an Ag/AgCl double junction reference electrode (made in Germany).

### Standard solutions

#### Stock standard solution of CLM

Clomipramine solution (1 × 10^− 2^ M) was prepared by transferring 90 mg of the standard CLM into a 25- mL volumetric flask followed by adding BRB buffer pH 3.0 to dissolve the powder, then the volume was completed to the mark using the same buffer.

#### Working standard solutions of CLM

Different working standard solutions of CLM with concentrations 1 × 10^− 5.3^ to 1 × 10^− 2^ M were freshly prepared in a series of 25- mL volumetric flasks and each time the volume was completed to the mark by BRB buffer pH 3.0.

#### Human plasma

The human plasma samples were obtained from VACSERA, Cairo, Egypt.

### Fabrication of sensor

#### Preparation of the Ion Selective membrane (ISM)

The ISM was formed by mixing 33.17 wt% PVC with 0.23 wt% KTpClPB, and 0.42 wt% of CX-4 and then dissolving them in 66.60 wt % of *o*-NPOE, followed by mixing with 6 mL of THF till getting a fully homogenous solution, specified as ISM(CX4).

#### Assembly of sensor modified with graphene nanocomposite

Graphene nanocomposite (Gr-NC) has been prepared as stated in the literature [29, 36]. The dispersion was used to form the graphene nanoparticles where 10 mg graphene nano-platelets were dispersed into 1.0 mL xylene and sonicated for 5 min. 95 mg PVC were mixed with 3 mL of THF and 0.2 mL *o*-NPOE plasticizer. The THF solution was added to the graphene dispersion and sonicated for 10 min in order to get the graphene nanocomposite [29]. To form the screen-printed carbon sensor, 10.0 µL Gr-NC dispersion was drop-casted onto the screen-printed carbon electrode and left to evaporate overnight at room temperature. Then, 10.0 µL of the ionophore-doped ion sensing membrane ISM(CX4) was drop-casted onto the C-SPE/Gr-NC and left to evaporate overnight to obtain sensor I: C-SPE/Gr-NC/ISM(CX4). To evaluate the role of Gr-NC on sensor performance, a control sensor (sensor II) was fabricated, with the exclusion of Gr-NC layer, sensor II: C-SPE/ISM(CX4).

### Sensors characterization

#### Sensors calibration

Each sensor separately was conjugated with the Ag/AgCl double-junction reference electrode. Then immersed the sensor into CLM drug solutions (1 × 10^− 5.3^−1 × 10^− 2^ M). The electromotive forces (*emf*) readings were allowed to equilibrate to within ± 1 mV then the reading was recorded. Calibration curves were constructed by plotting the recorded *emf* obtained from the sensor versus the corresponding − log concentrations of CLM. Then, the regression equations corresponding to each sensor were computed.

#### Estimation of the slope, response time and selectivity of the proposed sensors

The slope, response time and selectivity of the proposed sensors were evaluated in as per the IUPAC guidelines [37].

### Application to the dosage form

Ten tablets were weighed and crushed and 0.075 g (equivalent to one tablet) were transferred to 50- mL volumetric flask and the volume was completed to the mark with BRB buffer pH 3. 25 mL of the prepared solution was transferred to a 50- mL volumetric flask and the volume was completed with the buffer to get a claimed concentration 8.8 × 10^− 4^ M Clomipramine HCl (equivalent to 8 × 10^− 4^ M Clomipramine free base).

### Spiked plasma

A volume 0.5 mL blank plasma was placed in a falcon tube, spiked with fixed amount of CLM and a solutions equivalent to 1 × 10^− 4^ and 1 × 10^− 5^ CLM were mixed with plasma, pH adjusted to 3.0 using BRB buffer and spiked samples were analysed.

## Results and discussion

The combination of graphene and PVC nanocomposite provided a hydrophobic transducer layer on SC-ISE. The addition of calix[4]arene as an ionophore provided stable potential, and improved the stability, reproducibility, and selectivity of the fabricated electrodes. Solid-state transduction-based electrochemical sensor allows direct quantitation of the ions under investigation with minimal samples preparation. It is easy to handle and maintain in comparison with conventional liquid contact ion selective electrodes (LC-ISEs).

### Method development and optimization

#### Membrane composition

The ISM is primarily formed of a polymer matrix which acts as the mechanical support to the membrane. A plasticizer which dissolves the ion exchanger and plasticizes the membrane, in addition to modifying the lipophilicity of the membrane. An ion exchanger which is a hydrophobic ion with a charge opposite to that of the target ion, and an ionophore which selectively binds to the target ion. Furthermore, the plasticizer modifies the distribution coefficient (K_d_) of different species, so affects the performance characteristics of the electrode [38]. The ionophore calix[4]arene has higher binding strength to CLM, so it lowers the free energy of transfer of CLM from the aqueous phase to the ISM and leads to a selective signal of the electrode towards CLM. The composition of both sensors is presented in **Table S-1** (Supporting Information).

#### Potentiometric performance

As shown in Table 1, fabricated sensors showed Nernstian response towards CLM in the range of 1 x 10^− 2^ to 1 x 10^− 5.3^ M CLM. The response time is a crucial aspect for analytical procedures. The time required for the sensors to get to the final stable potential ± 1 mV was found to be 10 and 15 seconds for C-SPE/Gr-NC/ISM(CX4) and C-SPE/ISM(CX4), respectively as shown in Figure S-1 (Supporting Information) and Table 1.

**Table 1:** Electrochemical response characteristics of the fabricated sensors

Parameter	C-SPE/ISM(CX4)	C-SPE/Gr-NC/ISM(CX4)
Linear range	1 × 10^− 2^ to 1 × 10^− 5.3^ M	1 × 10^− 2^ to 1 × 10^− 5.3^ M
Slope (mV/decade)	-54.06	-55.42
Response time (s)	15	10
Working pH range	2–6	2–6
Stability (weeks)	4	4
LOD	1 × 10^− 5.39^ M	1 × 10^− 5.52^ M

#### pH effect on sensors performance

**Fig. 2:**
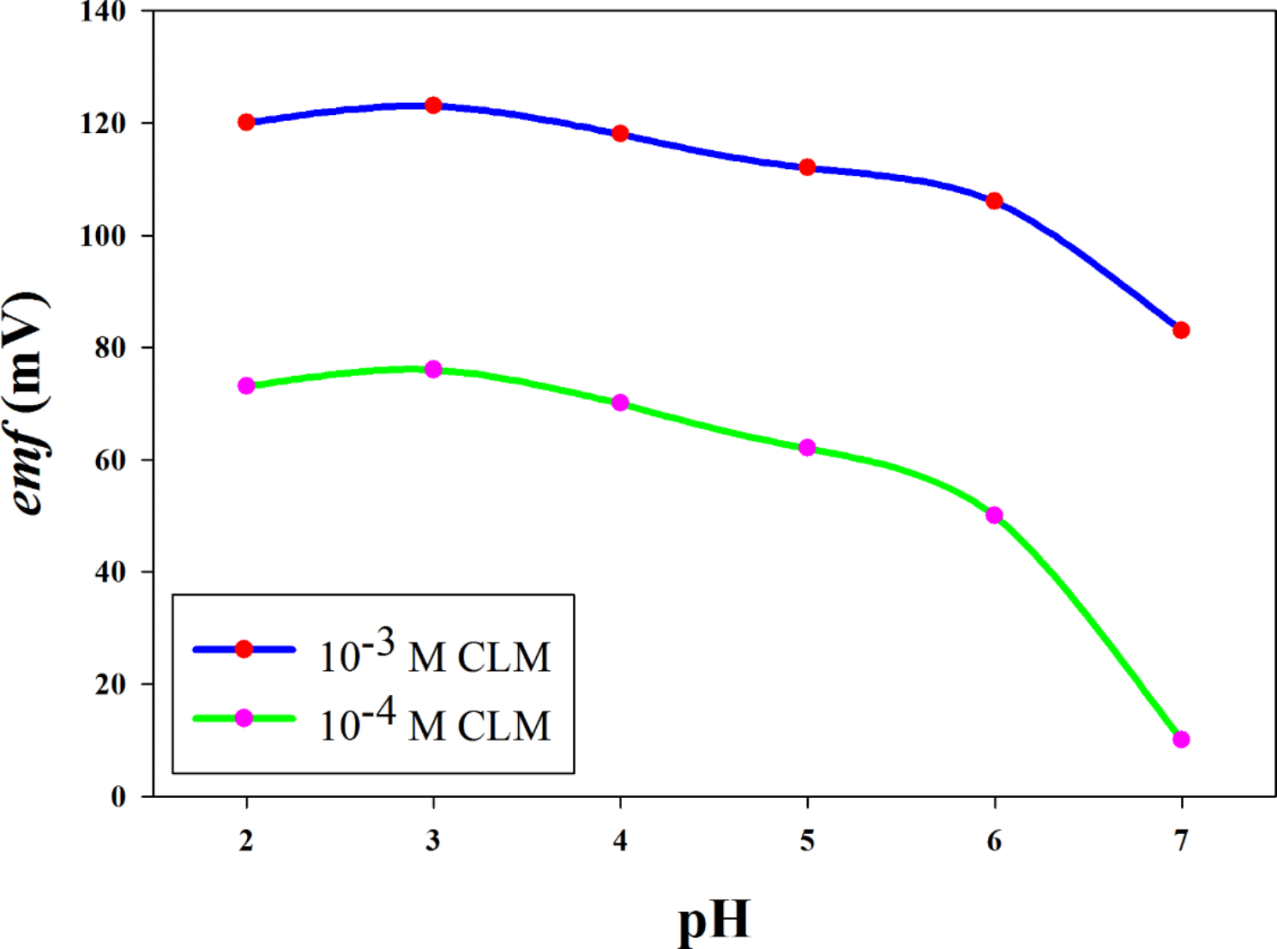
The effect of pH on the developed sensors

Optimum experimental conditions were studied for the quantitative applications of the fabricated potentiometric sensors. The potentiometric response has been reported by measuring *emf* of each sensor at different pH values in the range (3.0–9.0) using BRB buffer as shown in Fig. 2. It can be observed from the figure that the sensors have pH range 2.0–6.0, at this pH, CLM has positive charge (pK_a_ 9.42) [39] and can be exchanged at the membrane interface and produce *emf* changes.

#### Selectivity of the sensor

Selectivity coefficients of the fabricated sensors were computed using separate solution method and the following equation [30]:

$$-\log ({K^{pot}}_{A,B}) = \frac{{{E_1} - {E_2}}}{{2.303RT/{Z_A}F}} + \left( {1-\frac{{{Z_A}}}{{{Z_B}}}} \right)\log {a_A}$$

where ${K^{pot}}_{A,B}$ is the potentiometric selectivity coefficient, *E*_1_ is the potential measured in 10^− 4^ M CLM solution, *E*_2_ is the potential measured for 10^− 3^ M of the interfering ion. *Z*_*A*_ and *Z*_*B*_ are the charges of CLM and interfering ion, respectively, *a*_*A*_ is the activity of the drug and 2.303 *RT*/Z_*A*_*F* represent the slope of the investigated sensor (mV/concentration decade). The selectivity coefficients are presented in Table 2. The good selectivity of the sensors is due to the optimized combination of lipophilic cation exchanger, *o*-NPOE as a plasticizer with high dielectric constant, in addition to incorporation of the calixarene as supramolecular ionophore.

**Table 2:** Potentiometric selectivity coefficients (-log K) of the proposed sensors using the separate solutions method

Interfering ion (10^− 3^ M)	C-SPE/ISM(CX4)	C-SPE/Gr-NC/ISM(CX4)
Sodium chloride	-2.84	-3.44
Magnesium sulphate	-3.86	-4.09
Potassium chloride	-2.77	-3.35
Ammonium sulphate	-2.75	-3.38

### Method validation

**Fig. 3:**
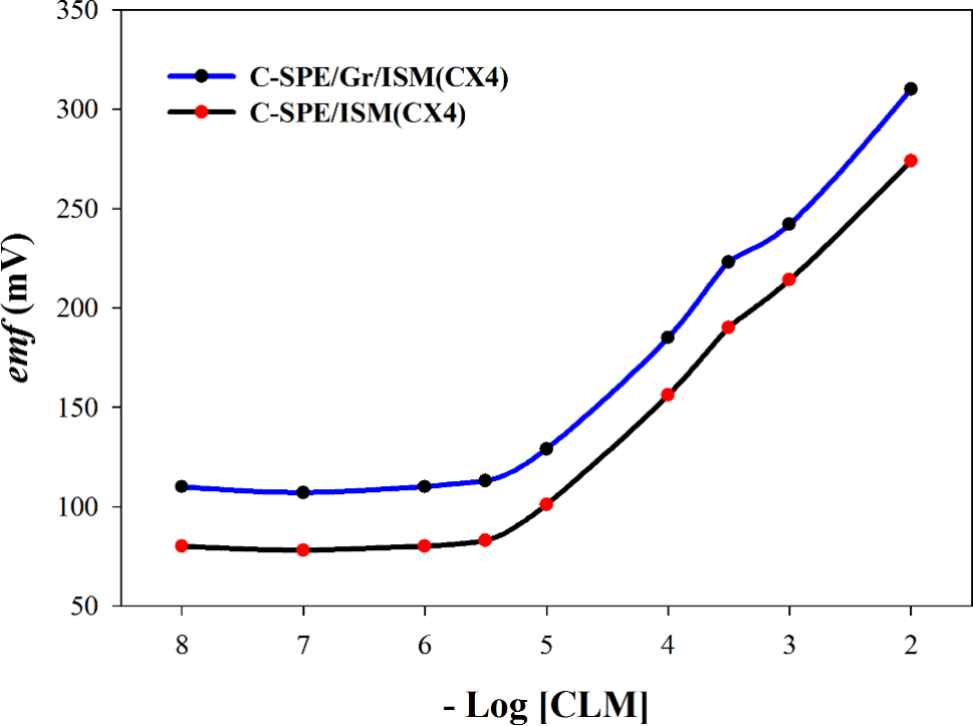
Profile of the potential in *emf* versus -log concentrations of CLM (M) obtained with the fabricated sensors

The potentiometric performance of the fabricated sensors was evaluated in the concentration range of 1 × 10^− 2^ to 1 × 10^− 5.3^ M. The electrodes revealed a good Nernstian response as shown in Fig. 3. Accuracy of the methods was assessed by covering three concentration levels in triplicates showing accepted percent recovery. Precision was examined and the results from applying repeatability and intermediated precision showed accepted RSD. The LOD and LOQ were calculated and a full validation sheet is presented in Table 3.

**Table 3:** Validation parameters of the developed electrochemical methods

Parameter	C-SPE/ISM(CX4)	C-SPE/Gr-NC/ISM(CX4)
Linearity		
Slope^a^	-54.06	-55.42
Intercept	411.23	382.11
R^2^	0.9932	0.9936
Range	1 × 10^− 2^ − 1 × 10^− 5.3^ M	
Accuracy^**b**^	100.60 ± 1.75	101.05 ± 1.10
Precision Repeatability^c^ Intermediate precision^c^	0.668 1.862	0.822 1.516

^a^ Average of three determinations

^b^ Accuracy results were done for (5 × 10^− 5^, 5 × 10^− 4^ and 5 × 10^− 3.5^ M) of CLM.

^c^ RSD for repeatability and intermediate precision were done for (10^− 5^, 10^− 4^ and 10^− 3^ M) of CLM.

### Pharmaceutical applications

#### Potentiometric assay of CLM in dosage form

The two sensors were successfully applied for the assay of CLM in Anafranil tablets^®^ showing no interference of excipients as represented on Table 4.

**Table 4:** Determination of CLM in pharmaceutical formulation and spiked plasma

Application	C-SPE/ISM(CX4)	C-SPE/Gr-NC/ISM(CX4)
Pharmaceutical Formulation (Anafronil tablets)® B.N: Y0021	99.48 ± 1.16^*^	102.10 ± 1.27^*^
Spiked plasma	102.65 ± 1.72^*^	100.26 ± 2.10^*^

^*^ Average of triplicate measurements

#### Potentiometric determination of CLM in spiked plasma

Plasma was spiked with 10^− 4^ and 10^− 5^ M CLM, and the sensors provided reliable results with acceptable accuracy and precision as shown in Table 4.

### Statistical comparison

The results obtained upon using the proposed sensors were compared to the results obtained upon applying the reported method [11]. The calculated student’s t-test and F test values indicated that there was no significant difference as shown in Table 5.

**Table 5:** Statistical analysis of the results obtained by the proposed sensors and those obtained by the reported method for the analysis of CLM in its pure powdered form

Parameter	C-SPE/ISM(CX4)	C-SPE/Gr-NC/ISM(CX4)	Reported method
Mean (Recovery %)	99.98	99.97	99.55
Variance	3.39	2.78	0.89
n	5	5	5
Student’s t-test (2.36)	0.46	0.49	**----**
F-test (6.38)	3.80	3.12	**----**

### Greenness assessment

**Fig. 4:**
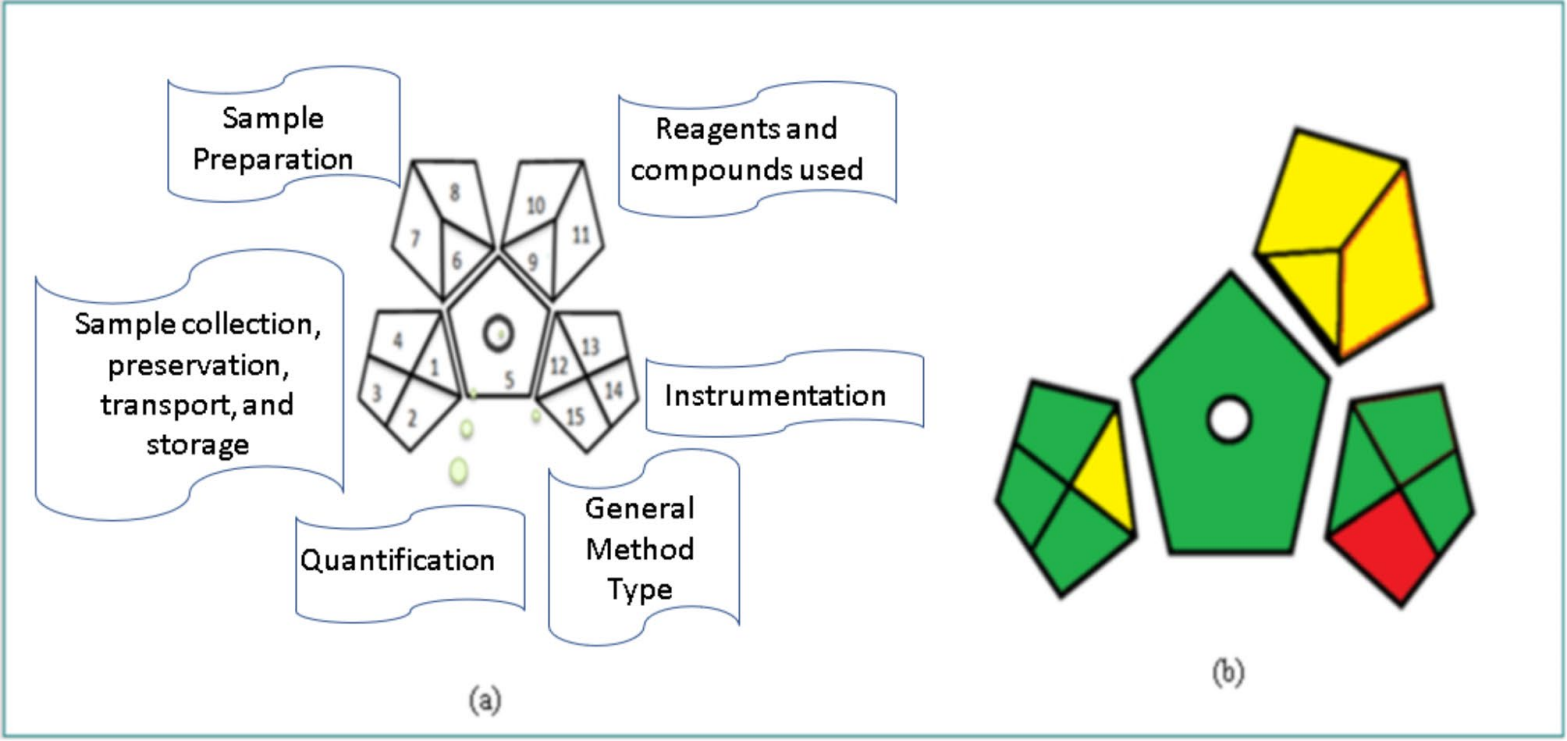
(a) GAPI original pentagram (b) GAPI pentagram for the ISE procedure

GAPI tool is a visual display of data composed of five pentagrams. These pentagrams assess the environmental impact of the method’s main stages as sample collection, preservation, transport and storage, general method type, sample preparation, reagents used and instrumentation. They are marked by red, yellow and green colours representing high, medium and low hazardous effect, respectively. There is no sample preparation step in the current method, so its representing pentagram is eliminated. The obtained GAPI pentagrams shown in Fig. 4 have a dominance of green colour suggesting low hazardous effect on the environment. However, the pentagram shows red colour representing that there was no waste treatment, and yellow colour equivalent to low reagents safety used; as BRB buffer and THF were incorporated in membrane fabrication have some health hazards.

**Fig. 5:**
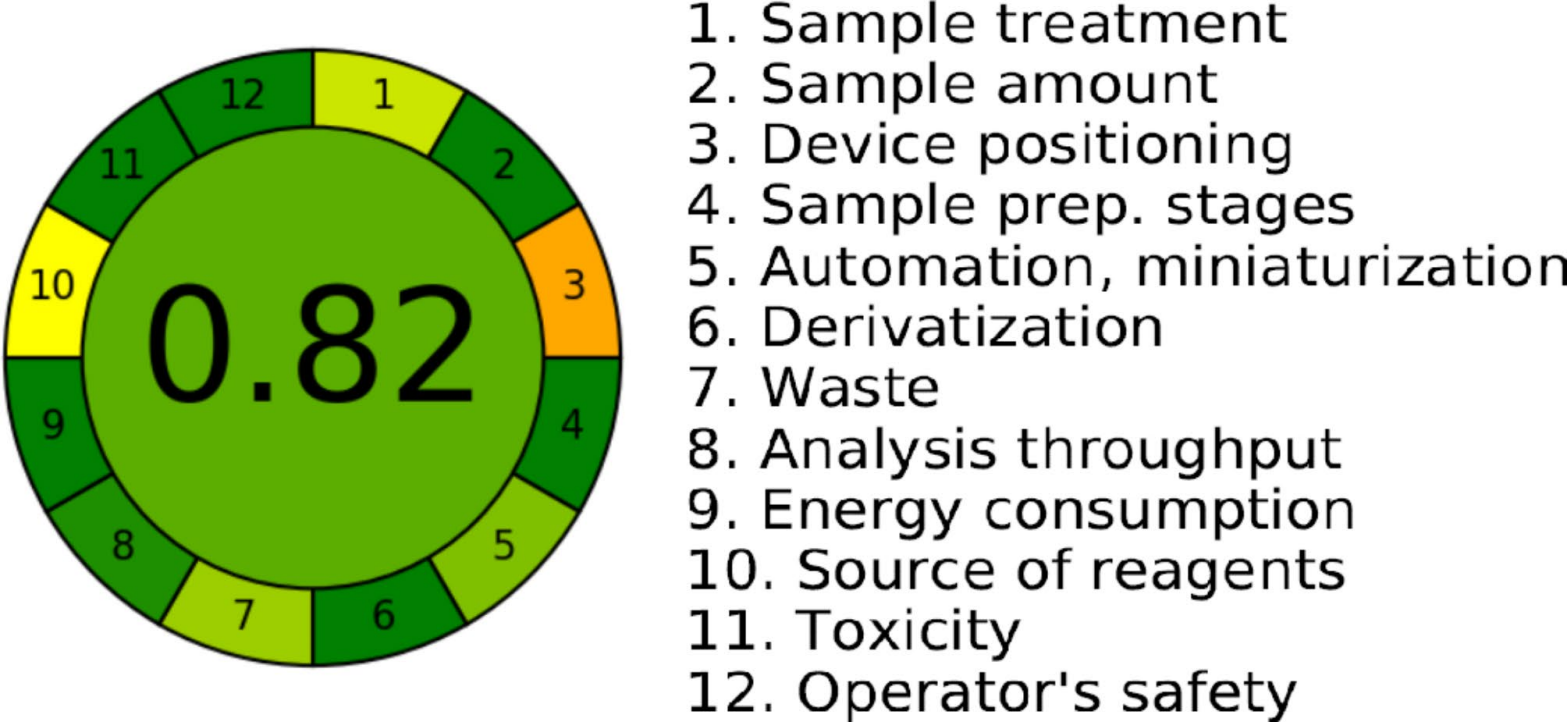
AGREE Greenness assessment score of the ISE procedures

The greenness analytical calculator; AGREE is the most recent greenness assessment tools. It is a user-friendly free software for assessing analytical operations. Assessment is based on twelve criteria, and alternative weights are applied to each one of them. Each one of the twelve input variables is converted to a numerical scale ranging from 0 to 1. The sum of the assessment results for each variable indicates the final greenness score. The result is a circular graph, showing the overall score in the centre. Values near 1 have dark green colour indicating that the tested procedure is more environmental friendly. The proposed ISE has an AGREE score of 0.82 and a middle green colour, Fig. 5, proving its low environmental impact and declaring that it is a green method.

### Water layer test

**Fig. 6:**
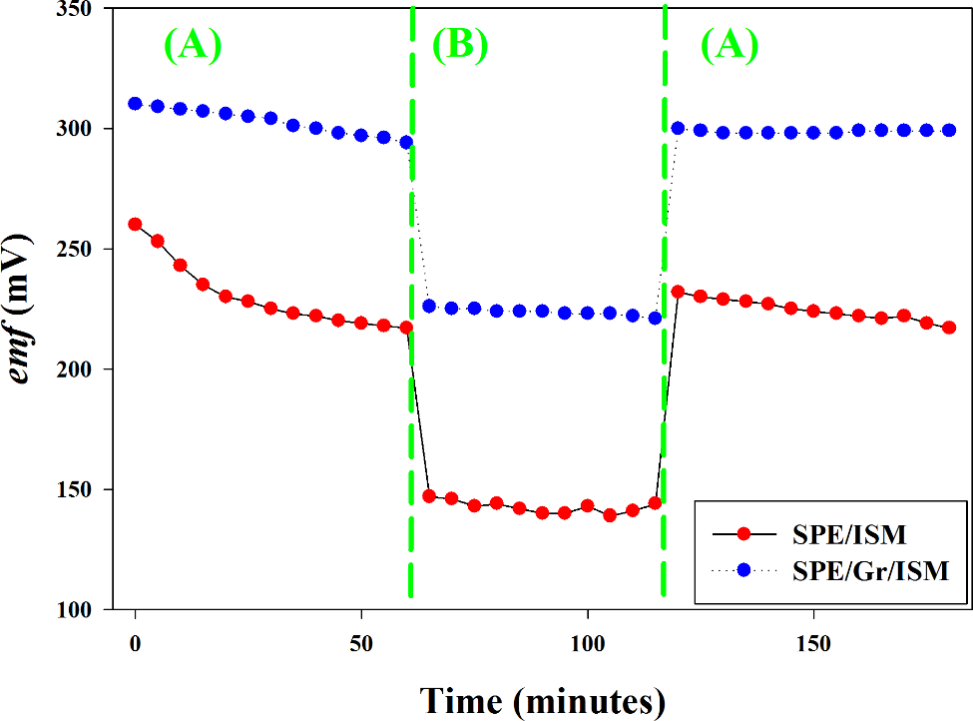
Potentiometric water layer test, the potential was recorded in solution (A) 10^− 3^ M CLM, solution (B) 10^− 2^ M Mebeverine and solution (A) 10^− 3^ M CLM again using SPE/ISM and SPE/Gr/ISM.

The test was conducted by subjecting the electrode to a high concentration of the interfering ion (mebeverine) and then estimate the drift in potential following testing a sample of lower concentration of the drug under investigation. The test was performed by measuring the potential of GR/ISM in 10^− 3^ M CLM solution then changing to 10^− 2^ M mebeverine solution then back to CLM solution. If a water layer was created under the membrane, its ionic structure would be changed due to ion fluxes and a potential drift would be noticed. The fabricated sensors showed a significant potential drift indicating the presence of an aqueous layer below the membrane, while GR/ISM sensor showed a more stable potential over time indicating that there was no water layer formed beneath the membrane due to the hydrophobicity of graphene as well as the conductivity and electrical capacity of the formed nanocomposite as shown in Fig. 6.

## Conclusion

This work was concerned with developing a green analytical method for the determination of CLM. The proposed SC-ISE was mixed with graphene nanocomposite transducer interlayer. The graphene layer succeeded in preventing the formation of an aqueous layer so resulted in a stable, reproducible standard potential besides the rapid response time. The reproducibility and stability of the proposed graphene-based sensor allows its application in CLM analysis in QC labs or in biological fluids to personalize the patient’s dosage.

## Electronic supplementary material

Below is the link to the electronic supplementary material.

Supplementary Material 1

## Data Availability

(ADM) The datasets used and/or analysed during the current study available from the corresponding author on reasonable request.

## List of Abbreviations

AGREE: Analytical GREEnness Metric Approach
ADHD: Attention deficit hyperactivity disorder
BP: British pharmacopoeia
BRB: Britton – Robinson buffer
C_max_: Maximum drug plasma concentration
C-SPE: Screen-printed carbon electrode
CX: Calixarene
CLM: Clomipramine
K_d_: Distribution coefficient
*Emf*: Electromotive forces
GAPI: Green Analytical Procedure Index
Gr-NC: Graphene nanocomposite
2-HP-β-CD: (2-hydroxypropyl)-β-cyclodextrin
ISM: Ion sensing membrane
IUPAC: International Union of Pure and Applied Chemistry
LC-ISEs: Liquid contact ion selective electrodes
LOD: Limit of detection
OCD: Obsessive-compulsive disorder
*o*-NPOE: *o*-nitrophenyl octyl ether
PVC: Polyvinyl chloride
KTpClPB: Potassium tetrakis(4-chlorophenyl)borate
QC: Quality control
RSD: Relative standard deviation
SC-ISEs: Solid-contact ion-selective electrodes
THF: Tetrahydrofuran
