# Supplementary material for: Determination of Clomipramine using eco-friendly solid-contact ionophore-doped potentiometric sensor
Source: BMC Chem. 2023 Mar 25;17(1):27. doi: 10.1186/s13065-023-00938-x (PMC10040110; doi:10.1186/s13065-023-00938-x)
Supplement: Supplementary file 1 — Supplementary Material 1 [file 13065_2023_938_MOESM1_ESM.docx]

**Supporting Information for**

## “Determination of Clomipramine Using Eco-friendly Solid-contact Ionophore-Doped Potentiometric Sensor”

Adel M. Michael^1^, Amr M. Mahmoud^2*^, Nesma M. Fahmy^1^

^1^Pharmaceutical Chemistry Department, Faculty of Pharmacy, Ahram Canadian University, Egypt

^2^Analytical Chemistry Department, Faculty of Pharmacy, Cairo University, Cairo, Egypt

^*^ Corresponding author email: [amr.bekhet@pharma.cu.edu.eg](mailto:amr.bekhet@pharma.cu.edu.eg)

Tel.: +201018338306

**Table S-1:** The composition of solid-contact ion-selective electrode sensors.

|  | **Sensor I**  **C-SPE/Gr-NC/ISM(CX4)** | **Sensor II**  **C-SPE/ISM(CX4)** |
| --- | --- | --- |
| **Polymer matrix** | PVC | PVC |
| **Plasticizer** | *o*-NPOE | *o*-NPOE |
| **Cation exchanger** | KTpClPB | KTpClPB |
| **Ionophore** | CX-4 | CX-4 |
| **Transducer layer** | Gr-NC | ----- |


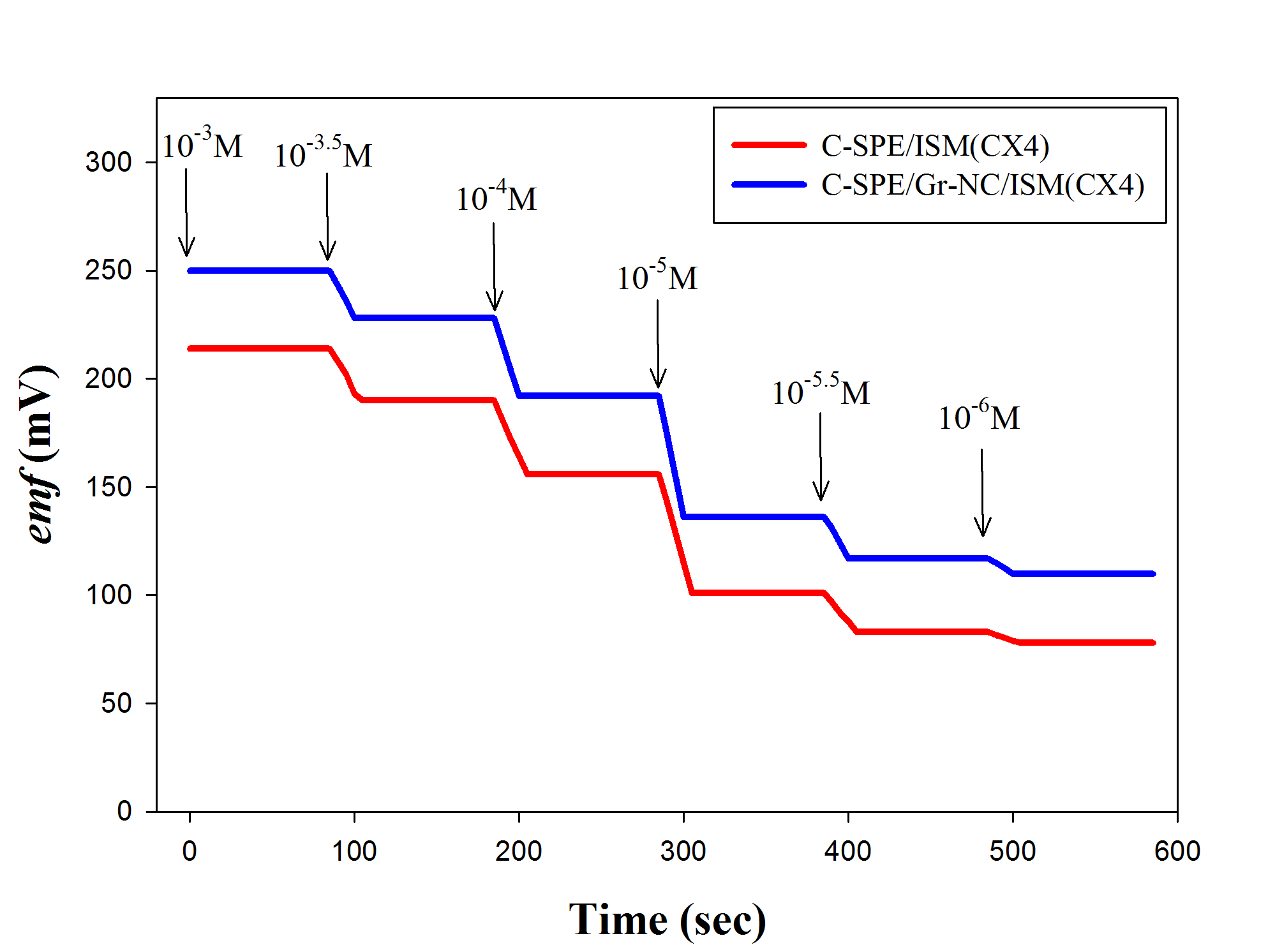


**Figure S-1.** Potential−time curves of C-SPE/ISM(CX4) and C-SPE/Gr-NC/ISM(CX4) sensors recorded for decreasing concentrations of CLM (1x10^-3^ M to 1x10^-6^ M).
